# Western Graduates

**Published:** 1833

**Authors:** 


### VIII. Western Graduates.

*Medical College of Ohio.* At the last Commencement, the degree of M. D. was conferred on 19 Alumni of the school; and the honorary degree of M. D. on Dr. William Williams, of Milford, Ohio, and Dr. John J. Moorman, of Virginia.

*Transylvania University.* At the last public Commencement, 67 young gentlemen received the degree of Doctor of Medicine.

